# Comparison of the Properties of Epoxy Resins Containing Various Trifluoromethyl Groups with Low Dielectric Constant

**DOI:** 10.3390/polym15132853

**Published:** 2023-06-28

**Authors:** Yurong Zhang, Haidan Lin, Kai Dong, Shasha Tang, Chengji Zhao

**Affiliations:** 1 Key Laboratory of High Performance Plastics, Ministry of Education, College of Chemistry, Jilin University, Changchun 130012, China; 2 Electric Power Research Institute, State Grid Jilin Electric Power Company, Changchun 130012, China

**Keywords:** trifluoromethyl substitution, low dielectric constant, hydrophobicity, thermal stability

## Abstract

A series of epoxy resins containing various trifluoromethyl groups were synthesized and thermally cured with diaminodiphenylmethane (DDM) and aminophenyl sulfone (DDS). All epoxy resins exhibited excellent thermal stability with the glass transition temperatures of above 128 °C and 5% weight loss temperatures of above 300 °C. DDS-cured epoxy resins possessed higher thermal stability than that of DDM-cured epoxy resins, while DDM-cured epoxy resins showed better mechanical, dielectric, and hydrophobic properties. Additionally, DDM-cured epoxy resins with different locations and numbers of trifluoromethyl groups showed flexural strength in the range of 95.55~152.36 MPa, flexural modulus in the range of 1.71~2.65 GPa, dielectric constant in the range of 2.55~3.05, and water absorption in the range of 0.49~0.95%. These results indicate that the incorporation of trifluoromethyl pendant groups into epoxy resins can be a valid strategy to improve the dielectric and hydrophobic performance.

## 1. Introduction

Epoxy resins have been widely applied in coatings, adhesives, advanced composites, and electronic materials [1,2,3,4,5] because of their excellent adhesive properties, electrical insulation properties, thermal and chemical stability, and convenient manufacturing process. Especially with the rapid development of the microelectronic industry, epoxy resins have attracted increasing attention as advanced electronic packaging materials [6,7,8,9,10,11], including the epoxy molding compound, epoxy electrically conductive silver paste, and die attach materials. Among various kinds of epoxy resins, diglycidyl ether of Bisphenol A (DGEBA) is one of the most representative commercial epoxy resins, which has occupied over 90% of the total production worldwide. However, the performance of DGEBA could not satisfy the severe demand for advanced electronic packaging materials, such as excellent thermal and mechanical properties, as well as low dielectric constant and loss. To fulfill the requirements of advanced electronic packaging technology, numerous contributions have been made to preparing alternative epoxy resins to DGEBA with superior properties [12,13,14]. 

It has been demonstrated that the modification on the backbone or side chains of polymers by introducing trifluoromethyl moieties has become one of the most efficient strategies to endow the materials with low dielectric constant, enhanced mechanical and thermal properties, as well as hydrophobic performance [15,16,17]. The reduced dielectric constant is attributed to the low polarizability and large free volume of trifluoromethyl groups [18], while the improved stability and hydrophobic characteristics are due to the high bond energy of C-F. Hence, novel epoxy resins containing trifluoromethyl groups have attracted great interest in the electronic packaging filed. For example, Lee et al. investigated the substitution effects of methyl and trifluoromethyl groups on the performance of epoxy resins [19]. The results indicate that the trifluoromethyl group-substituted epoxy resins exhibited enhanced thermal resistance and a lower dielectric constant than that of the methyl group-substituted one. Tao [20] and Ge [21] synthesized epoxy resins with trifluoromethyl groups. Compared to commercial DGEBA, fluorinated epoxy resins possessed a lower dielectric constant (less than 3.3) and water absorption and excellent mechanical, electrical, and thermal properties [22]. In our previous work, we prepared a novel kind of fluorinated epoxy resin (3-TFMEP) and modified it with inorganic nanofiller boron nitride [23,24]. The cured 3-TFMEP resins showed good thermal stability and hydrophobicity, low dielectric constant, as well as good mobility of inorganic fillers. These results indicate that fluorinated epoxy resins have good potential in the application of electronic packaging and microelectronics.

In this study, in order to figure out the relationship between the structure of fluorinated epoxy resins and their comprehensive properties as electronic packaging materials, a series of novel epoxy resins containing different positions and amounts of trifluoromethyl groups (FER) in the side chains were synthesized and then cured with aromatic amine 4, 4′-diaminodiphenylmethane (DDM) and 4, 4′-diaminophenyl sulfone (DDS). The curing kinetics of FER was studied by the iso-thermal DSC method. The thermal stability and mechanical and dielectric properties, together with water absorption, of fluorinated epoxy resins were investigated and compared in detail. The results show that the molecular structure of FER and the curing agent played a vital role in determining the performance and hydrophobic characteristics of cured epoxy resins. Moreover, this work provides a strategy to obtain alternative epoxy resins with a low dielectric constant and good moisture resistance by adjusting the position and amount of trifluoromethyl groups in the side chains. 

## 2. Experimental

### 2.1. Materials

3,5-Bis(trifluoromethyl)aniline (≥97.0%), 3-aminobenzotrifluoride (≥99.0%), 4-aminobenzotrifluoride (≥99.0%), and DDS (≥99.0%) were purchased from Energy Chemical, Shanghai, China. p-Benzoquinone (≥97.0%) was obtained from Aladdin chemistry Co., Ltd, Shanghai, China. Sodium nitrite, hydrochloric acid (37%), sodium hydrogen carbonate, toluene, epichlorohydrin, sodium hydroxide, acetone, and ethanol were all analytical pure and obtained from Beijing chemical company, Beijing, China. Zinc powder, tetrabutylammonium bromide, potassium acid phthalate, DDM, methyl red, and phenolphthalein were all analytical pure and purchased from Sinopharm Chemical Reagent Co., Ltd., Shanghai, China. All reagents were used as received without further purification.

### 2.2. Synthesis of Phenylhydroquinones Containing Trifluoromethyl Groups (FPQs)

**Figure 1:**
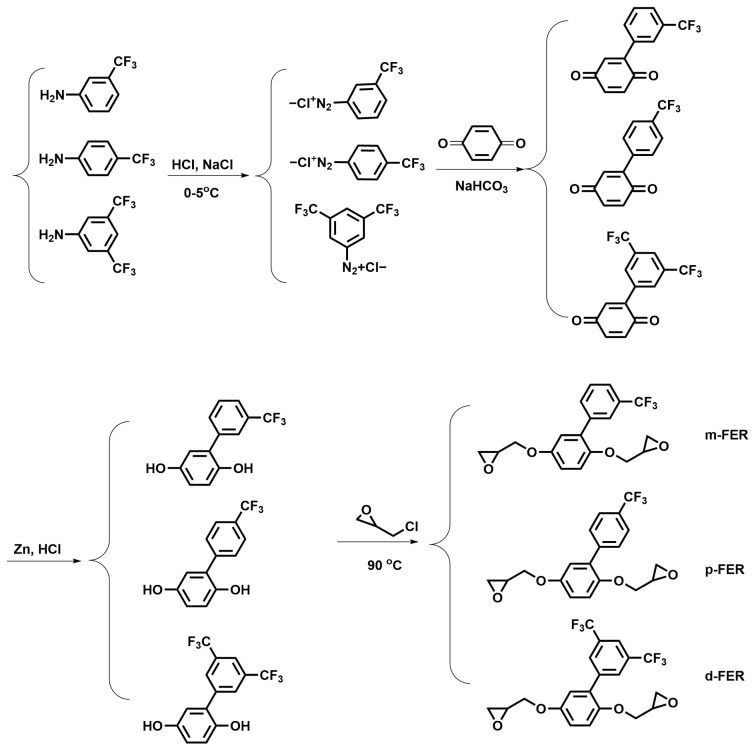
The synthesis of phenylhydroquinones and epoxy resins containing various locations and numbers of trifluoromethyl substitutes.

As shown in Figure 1 *m*-FPQ, *p*-FPQ, and *d*-FPQ containing various locations and numbers of trifluoromethyl substituents on the phenylhydroquinones were synthesized through diazo reaction and followed by a Meerwein arylation reaction, respectively. Aniline containing trifluoromethyl groups (3-aminobenzotrifluoride, 4-aminobenzotrifluoride or 3,5-bis(trifluoromethyl)aniline, 0.1 mol), hydrochloric acid (33 mL), and deionized water (33 mL) were added into a beaker equipped with a mechanical stirrer and a thermometer. After cooling to 2 °C, sodium nitrite aqueous solution (30 wt.%, 70 g) was added dropwise into the beaker, and the temperature of the system was kept between 0 and 3 °C. The mixture was stirred for 30 min, and a transparent solution was obtained from filtration. Then the solution was added dropwise into a mixture of *p*-benzoquinone (0.08 mol), sodium hydrogen carbonate (0.03 mol), and deionized water (100 mL). The mixture was stirred at 8~10 °C for 4 h. After filtration, the precipitate was washed with deionized water. The obtained yellow solid was dried at room temperature.

The above yellow solid (0.05 mol), zinc powder (0.15 mol), and deionized water (60 mL) were added into a three-neck flask equipped with a mechanical stirrer, a dropping funnel, and a condenser. The mixture was heated to 90 °C, and then hydrochloric acid (35 mL) was added dropwise into the flask. The reaction mixture was allowed to reflux for 4 h and then filtered. The precipitate was recrystallized from toluene and dried in a vacuum oven for 24 h.

*m*-FPQ: yield: 80%, melting point: 88 °C. ^1^H-NMR (500 MHz, DMSO-d6, δ) δ 9.04 (s, 1 H), 8.90 (s, 1H), 7.92–7.73 (m, 2 H), 7.72–7.53 (m, 2 H), 6.79 (d, *J* = 8.6 Hz, 1 H), 6.72 (d, *J* = 2.8 Hz, 1 H), 6.65 (dd, *J* = 8.6, 2.9 Hz, 1 H). Elem. Anal. Calcd. for C_13_H_9_F_3_O_2:_ C, 61.42%; H, 3.57%; O, 12.59%. Found C, 61.51%; H, 3.44%; O, 12.72%. 

*p*-FPQ: yield: 80%, melting point: 111 °C. ^1^H-NMR (500 MHz, DMSO-d6, δ) δ 9.01 (s, 1 H), 8.88 (s, 1 H), 7.74 (s, 4 H), 6.79 (d, *J* = 8.6 Hz, 1 H), 6.72 (d, *J* = 2.9 Hz, 1 H), 6.65 (dd, *J* = 8.6, 3.0 Hz, 1 H). Elem. Anal. Calcd. for C_13_H_9_F_3_O_2:_ C, 61.42%; H, 3.57%; O, 12.59%. Found C, 61.34%; H, 3.56%; O, 12.60%.

*d*-FPQ: yield: 75%, melting point: 112 °C. ^1^H-NMR (500 MHz, DMSO-d6, δ) δ 9.15 (s, 1 H), 8.85 (s, 1 H), 8.08 (s, 2 H), 7.92 (s, 1 H), 6.73 (d, *J* = 8.9 Hz, 2 H), 6.61 (dd, *J* = 8.6, 2.8 Hz, 1 H). Elem. Anal. Calcd. for C_14_H_8_F_6_O_2:_ C, 52.19%; H, 2.50%; O, 9.93%. Found C, 52.22%; H, 2.43%; O, 10.02%.

### 2.3. Synthesis of Epoxy Resin Monomers Containing Trifluoromethyl Groups (FERs)

According to the location and number of trifluoromethyl substituents on the FERs, *m*-FER, *p*-FER, and *d*-FER were prepared by a similar procedure. Taking *m*-FER as an example, the procedure is described as the following. A mixture of *m*-FPQ (0.1 mol), epichlorohydrin (157 mL), and tetrabutylammonium bromide (1.93 g) was mixed in a three-neck flask at 90 °C for 6 h. The excess epichlorohydrin was removed at a reduced pressure. Then, toluene (160 mL) and sodium hydroxide (48.67 g, 30 wt.% solution) were added to the mixture, and the reaction was kept at 90 °C for another 3 h. Then, the mixture was washed with deionized water at room temperature until the water was neutral. The toluene and water were distilled under reduced pressure. The product was dried in the oven at 80 °C for 24 h.

*m*-FER: yield: 85%, liquid at room temperature, epoxy value: 0.52. ^1^H-NMR (500 MHz, DMSO-d6, δ) δ 7.44 (d, *J* = 8.0 Hz, 2 H), 7.23 (d, *J* = 8.0 Hz, 2 H), 7.09–6.98 (m, 1 H), 6.96–6.84 (m, 2 H), 4.32 (dd, *J* = 11.4, 2.5 Hz, 1 H), 4.24 (dd, *J* = 11.4, 2.3 Hz, 1 H), 3.37–3.28 (m, 1 H), 3.22 (td, *J* = 6.3, 2.5 Hz, 1 H), 2.79 (m, 2 H), 2.71–2.58 (m, 2 H). Elem. Anal. Calcd. for C_19_H_17_F_3_O_4:_ C, 62.29%; H, 4.68%; O, 17.47%. Found C, 61.88%; H, 4.70%; O, 17.39%.

*p*-FER: yield: 87%, melting point: 115 °C, epoxy value: 0.52. ^1^H-NMR (500 MHz, DMSO-d6, δ) δ 7.78 (s, 4H), 7.15–7.07 (m, 1 H), 7.04–6.94 (m, 2 H), 4.33 (m, 2 H), 3.85 (m, 2 H), 3.36–3.32 (m, 1 H), 3.25 (m, 1 H), 2.86–2.81 (m, 1 H), 2.81–2.74 (m, 1 H), 2.71 (m, 1 H), 2.62 (m, 1 H). Elem. Anal. Calcd. for C_19_H_17_F_3_O_4:_ C, 62.29%; H, 4.68%; O, 17.47%. Found C, 62.03%; H, 4.58%; O, 17.35%.

*d*-FER: yield: 80%, melting point: 110 °C, epoxy value: 0.44. ^1^H-NMR (500 MHz, DMSO-d6, δ) δ 8.24 (s, 2 H), 8.07 (s, 1 H), 7.15 (t, *J* = 6.0 Hz, 2 H), 7.05 (dd, *J* = 9.0, 3.0 Hz, 1 H), 4.35 (m, 2 H), 3.94–3.85 (m, 2 H), 3.34 (dd, *J* = 6.7, 2.6 Hz, 1 H), 3.22 (td, *J* = 6.6, 2.6 Hz, 1 H), 2.80 (m, 2 H), 2.73–2.56 (m, 2 H). Elem. Anal. Calcd. for C_20_H_16_F_6_O_4:_ C, 55.31%; H, 3.71%; O, 14.73%. Found C, 55.29%; H, 3.70%; O, 14.68%.

### 2.4. Preparation of the Cured Epoxy Resins

The epoxy resins were cured with two kinds of curing agents, DDM and DDS, respectively. According to the epoxy values, the stoichiometric amounts of DDM and DDS were added to the epoxy mixtures with acetone as the solvent. Then, the mixtures were stirred for 30 min at room temperature. The solution was poured into a mould. The mould was dried in a vacuum oven at 40 °C for 12 h to remove the solvent. The onset curing temperature (*T*_i_), the peak curing temperature (*T*_p_), and the end-set curing temperature (*T*_e_) of epoxy resins are listed in Table S1. The curing temperature of epoxy resins was determined by the iso-thermal differential scanning calorimetry (DSC) measurement and the linear fitting of the *T*_i_, *T*_p_, and *T*_e_ [25]. Generally, the epoxy resins were cured at *T*_i_ for 2 h, at *T*_p_ for 2 h, and then at *T*_e_ for 1 h.

### 2.5. Characterization

^1^H-NMR spectra were measured on a 510 spectrometer (Bruker, Fällanden, Switzerland) at 500 MHz using deuterated dimethyl sulfoxide (DMSO-d_6_) as the solvent and tetramethylsilane (TMS) as the standard. The elemental contents of C, H, and O were determined using an elemental analyzer (model Vario EL cube, Elementar, Langenselbold, Germany). The epoxy value was determined via the acetone-hydrochloride method, and methyl red was used as the indicator. The epoxy value was calculated as the mean of the three tests. The cure kinetics and glass transition temperature (*T*_g_) of the cured epoxy resins were measured by using DSC (Q20, TA instrument, New Castle, DE, USA) under nitrogen at a flow rate of 50 mL/min. Thermogravimetry analysis (TGA) was performed on the Perkin-Elmer Pyris1 thermogravimetric analyzer (Waltham, MA, USA) from 80 to 800 °C at a heating rate of 10 °C/min under a nitrogen atmosphere. The flexural properties of samples were evaluated at room temperature on a universal tensile testing machine (AG-I 20 KN, Shimadzu, Japan) at a speed of 1 mm/min. The dielectric constant and dielectric loss of epoxy resins were measured at room temperature by a 4292 precision impedance analyzer (Agilent, Santa Clara, CA, USA) in a range of frequencies from 1 kHz to 1 MHz. Water absorption was measured by immersing epoxy resins in water at 80 °C for 48 h, which was then wiped with tissue paper and weighted to determine the water absorption value. The water contact angle was measured using a contact angle goniometer (Drop shape Analysis DSA 30, Kruss, Hamburg, Germany) at room temperature.

## 3. Results and Discussion

### 3.1. Characteristics of Epoxy Resins Containing Trifluoromethyl Groups

**Figure 2:**
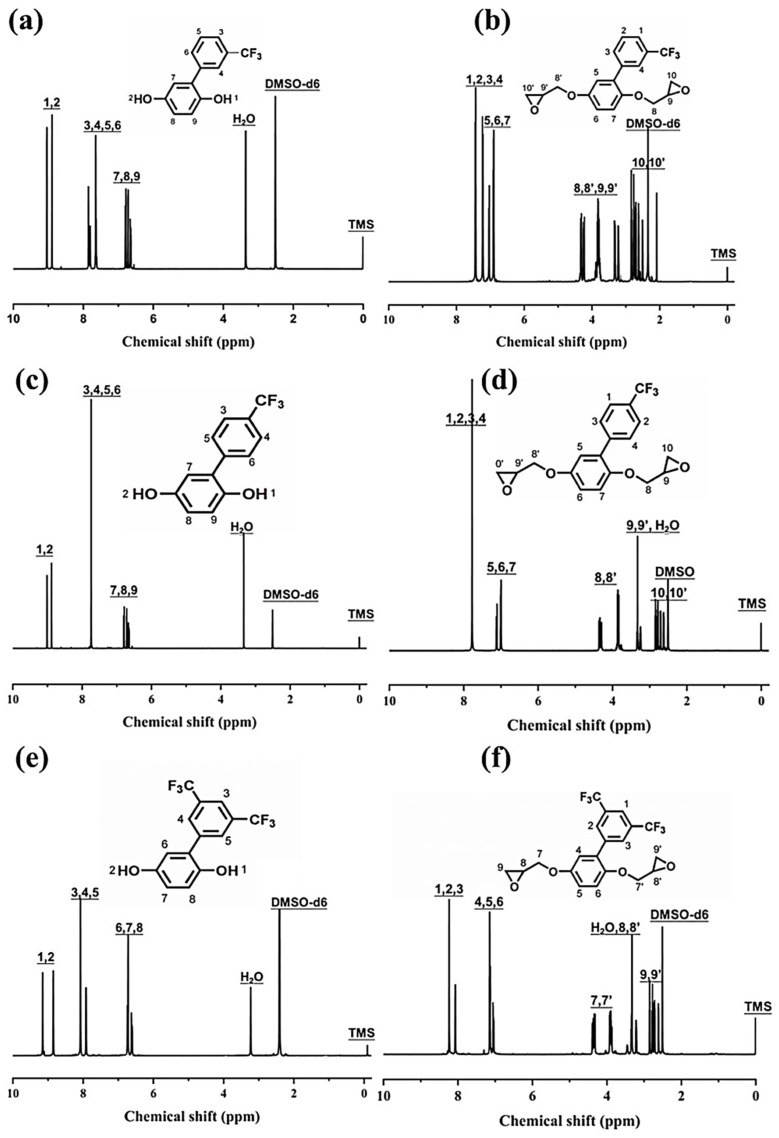
The ^1^H-NMR spectra of phenylhydroquinones and epoxy resin monomers containing various trifluoromethyl groups. *m*-FPQ (**a**); *m*-FER (**b**); *p*-FPQ (**c**); *p*-FER (**d**); *d*-FPQ (**e**); *d*-FER (**f**).

The structure of FPQs and FERs was confirmed by ^1^H-NMR and elemental analysis, as shown in Figure 2. For the FPQs, the peaks at 9 ppm are assigned to the phenolic hydroxyl group, and peaks between 6 and 8.5 ppm are attributed to the protons on the aromatic ring. For the FERs, the signals for the phenolic hydroxyl group disappear, while the peaks corresponding to the protons on the methene and methyne of epoxide groups of FERs appear at the chemical shifts between 2.5 and 4.5 ppm. The peak integrals and the chemical shifts of all protons were consistent with the chemical structure of FERs. The results of the elemental analysis were in good agreement with the calculated values. Furthermore, the titrated epoxy values of *m*-FER, *p*-FER, and *d*-FER were 0.52, 0.52, and 0.44, which were close to the theoretical epoxy values. All these results indicate that FERs were synthesized successfully.

### 3.2. Curing Kinetic Analysis

**Figure 3:**
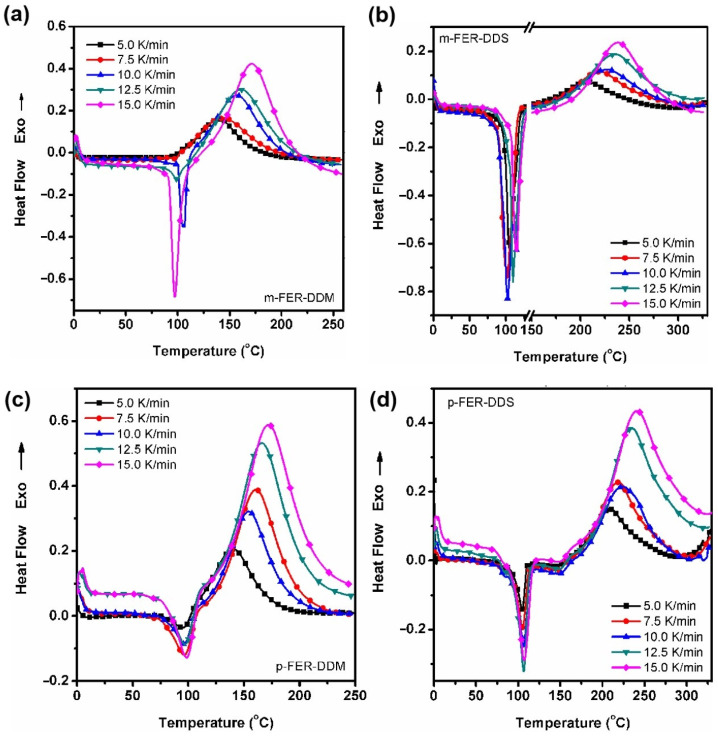

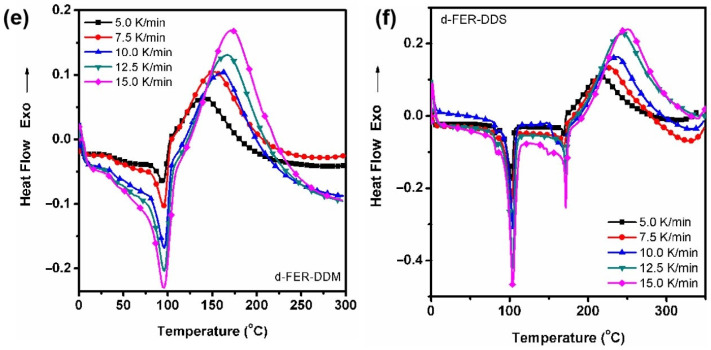
Non−isothermal DSC curves of epoxy resins at different heating rates, (**a**) *m*−FER-DDM, (**b**) *m*−FER-DDS, (**c**) *p*−FER-DDM, (**d**) *p*−FER-DDS, (**e**) *d*−FER-DDM, and (**f**) *d*−FER-DDS.

The investigation of curing kinetics can be helpful to produce high-performance thermosetting polymers. The curing behaviors of FERs were investigated by the iso-thermal DSC method using DDM and DDS as the curing agents at different heating rates (i.e., 5.0, 7.5, 10.0, 12.5, and 15.0 °C/min) [26]. The dynamic DSC thermographs of epoxy resins are depicted in Figure 3. For the DDM-cured FERs, there is only one endothermic peak in their DSC curves, which is the eutectic point of DDM (89 °C) and FERs (*m*-FER: liquid; *p*-FER: 115 °C; *d*-FER: 110 °C). This phenomenon was also observed from the DSC curves of DDS-cured *m*-FER and *p*-FER with one endothermic peak. However, there are two endothermic peaks (110 and 180 °C) in the DSC curves of DDS-cured *d*-FER. The corresponding temperatures of endothermic peaks are in accordance with the independent melting points of the immiscible mixture (*d*-FER and DDS). Furthermore, there is only one exothermic peak in the curves for all samples. Similar to the published work [27], the temperature for the exothermic peak (*T*_p_) in their DSC curves shows an increasing tendency as the heating rate increases. Table S1 summarizes the *T*_i_, *T*_p_, and *T*_e_ of DDM and DDS-cured fluorinated epoxy resins. Compared to DDM-cured FERs, all the *T*_i_, *T*_p_, and *T*_e_ of DDS-cured samples are much higher. It can be attributed to the electron-withdrawing sulfone in DDS, which results in the weaker reactivity of DDS and higher curing temperature. Moreover, all the *T*_i_, *T*_p_, and *T*_e_ of DDS-cured *d*-FER samples are higher than those of other kinds of FERs. This is because the introduction of two trifluoromethyl substituents on the phenyl side chain decreases the reactivity between FERs and DDS. 

In this study, the overall activation energy (*E*_a_) of FERs was determined by the Kissinger method [28]. The following equation is based on the Kissinger method:

(1) $$\frac{d[\ln({\alpha /T}_{p}^{2})]}{d(1/T_{p})}=\frac{-E_{a}}{R}$$

where α is the heating rate, and *R* is the ideal gas constant, 8.314 J/(mol·K). 

**Figure 4:**
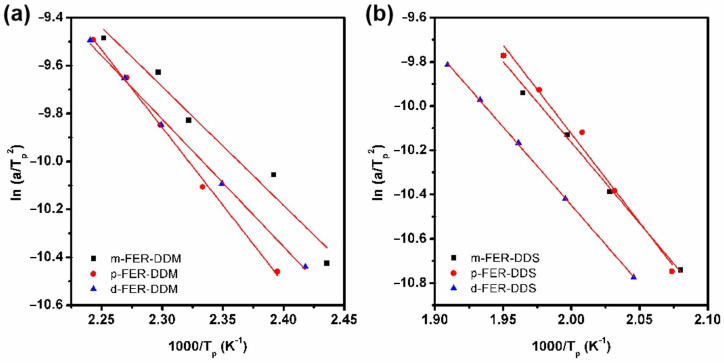
Plots for the determination of the *E*a by the Kissinger method in different curing reactions, (**a**) FER−DDM system, and (**b**) FER−DDS system.

Therefore, the *E_a_* of FERs can be calculated from the slope of the linear-fitting curve of ln(α/T*p*^2^) versus 1/*T_p_*, as displayed in Figure 4. The obtained *E_a_* of DDM-cured FERs are in a range of 41.1~53.9 kJ/mol, while the calculated *E_a_* of DDS-cured FERs are in a range of 58.8~66.6 kJ/mol. The higher *E_a_* of the DDS system implies that the DDS is a curing agent with weaker reactivity, which requires higher curing temperatures. Furthermore, the nth order of the curing reaction can be calculated from Crane’s equation [29]:

(2) $$\frac{d(\ln\alpha )}{d(1/T_{p})}=-(\frac{E_{a}}{nR}+2T_{p})$$

It was found that all these curing epoxy systems possessed a similar reaction order (*n*) of 0.92 ± 0.02, indicating the curing reaction mechanism between FERs and curing agents (DDM and DDS). The curing process was accomplished by the epoxy ring-opening reaction between the epoxy group and the primary amino group of curing agents.

### 3.3. Thermal Properties of Epoxy Resins

The *T*_g_ values of cured FERs were determined by DSC measurement at a heating rate of 10 °C/min, and the results are listed in Table 1. The DDS-cured FERs show *T*_g_ values in the range of 160~185 °C, which are around 30~40 °C higher than those of DDM-cured FERs. The sulfone moiety in DDS leads to much stronger intermolecular interactions, which confines the movement of the polymer chain segment. Among these cured resins, *p*-FER exhibits the highest *T*_g_ value of 185 °C, which is even higher than that of *d*-FER with two −CF_3_ substitutions (178 °C). The incorporation of two trifluoromethyl substitutions may result in higher electrostatic repulsion than intermolecular attraction, thus increasing the polymer chain spacing and then leading to a slightly lower *T*_g_.

**Figure 5:**
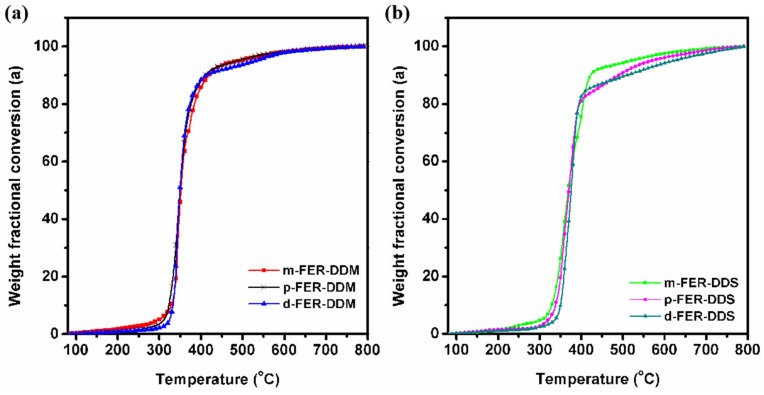
The weight fractional conversation (α) of epoxy resins, (**a**) FER-DDM system, and (**b**) FER-DDS system.

The thermal stability of DDM and DDS-cured FERs was evaluated by TGA. As shown in Figure S1, it can be observed that all the cured FERs exhibit a single-step thermal degradation pattern starting from above 300 °C, indicating that they possess good thermal stability below 300 °C. Furthermore, the weight fraction conversion (α) was defined as,

(3) $$\alpha =\frac{W_{0}-W_{t}}{W_{0}-W_{\infty }}$$

where W_0_, W_t_, and W_∞_ are the initial weight, the weight at a given temperature, and the final weight of the sample recorded by TGA As shown in Figure 5, all the cured FERs were stable below 300 °C, and as the heating temperature increased, the samples started to decrease their weight dramatically. When the temperature was above 450 °C, the weight loss of epoxy resins slowed down again. The relative thermal stability of cured FERs was compared by the decomposition temperatures at 5% and 10% of weight loss (*T*_d5_ and *T*_d10_), as presented in Table 2. These values are comparable to those of commercial DGEBA as reported [20]. Due to the existence of two −CF_3_ substitutions on the phenyl side chain, the cured *d*-FER samples exhibited higher *T*_d5_ and *T*_d10_ values than those of *m*-FER and *p*-FER samples. Moreover, DDS-cured epoxy resins possessed better thermal stability than DDM-cured samples, owing to the strong inter-chain interactions deriving from sulfone moiety with high polarization. As a result, the thermal stability of epoxy resins can be enhanced by introducing trifluoromethyl groups and increasing the inter-chain interactions.

### 3.4. Flexural Properties

To determine the mechanical flexibility of these cured FERs for electronic packaging application, we carried out a 3-point bending test on their rectangular-shaped samples and then obtained the flexural stress and modulus [11,30]. The flexural properties of these cured FERs are listed in Table 2. The flexural moduli of DDM-cured epoxy resins (1.71~2.65 GPa) are higher than those of DDS-cured ones (1.47~1.98 GPa), while the flexural strength values of DDM-cured epoxy resins (95.55~152.36 MPa) are also higher than those of DDS-cured samples (42.30~118.85 MPa). It might be because the curing temperatures of DDS-cured samples are much higher than that of DDM-cured ones. According to the study by Ge [21], higher curing temperatures might lead to worse mechanical performance, owing to the thermal degradation of epoxy resins during the curing process. Among these cured FERs, both *d*-FER samples cured by DDM and DDS exhibited moderate flexural strengths (127.00 and 118.85 MPa). This was possibly attributed to the rigid biphenyl scaffold in the chains and two polar −CF_3_ groups in the side chain.

### 3.5. Water Absorption and Contact Angle

It is crucial to possess low water absorption for low-*k* materials which are applied in microelectronics [31]. The water absorption values of FERs at 80 °C are shown in Table 2. Compared with DDS-cured epoxy resins, DDM-cured samples showed lower water absorption. This might be owing to the sulfone moiety in the main-chain of DDS, which possesses strong polarity. Additionally, the cured *d*-FER samples exhibited much lower water absorption values than those of the others. Among them, DDM-cured *d*-FER with two −CF_3_ substitutions on the phenyl side chain possessed the lowest water absorption of 0.49% due to the low surface energy and hydrophobic characteristic of trifluoromethyl groups [32]. This water absorption is also lower than that of conventional DDM-cured DGEBA (0.98) [20]. The results indicate that water absorption could be notably decreased by introducing more trifluoromethyl groups into molecule structure.

**Figure 6:**
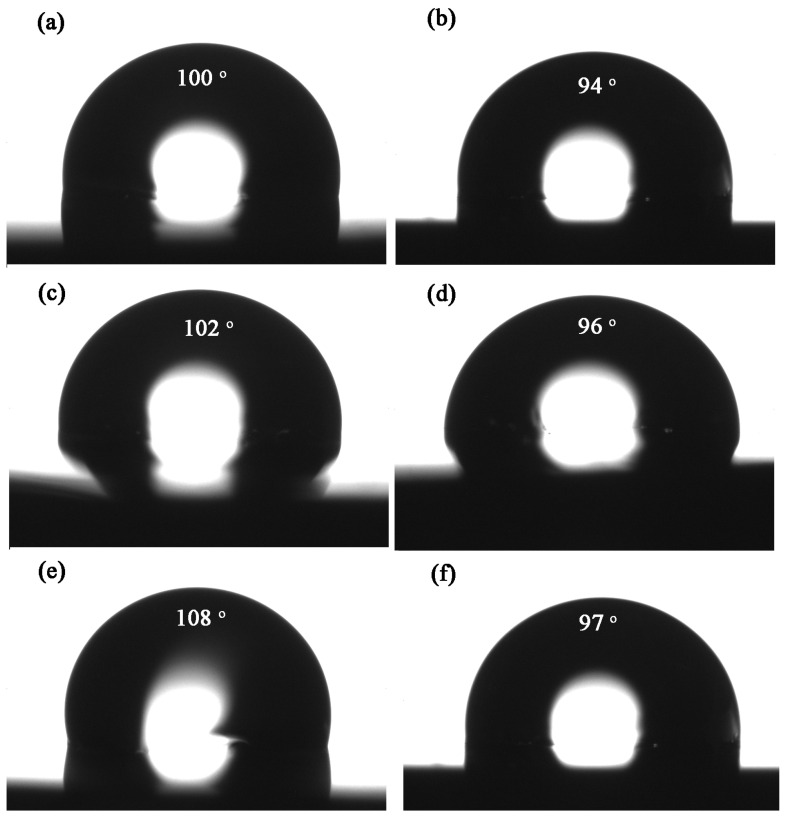
Water contact angle of FERs, (**a**) *m*-FER-DDM, (**b**) *m*-FER-DDS, (**c**) *p*-FER-DDM, (**d**) *p*-FER-DDS, (**e**) *d*-FER-DDM, and (**f**) *d*-FER-DDS.

In order to investigate the nature of water absorption, the water contact angle of these cured FERs was measured, and the results are shown in Figure 6. Compared to DDM-cured DGEBA with a contact angle of only 88°, all these cured FERs possess contact angle values above 90°. Furthermore, in comparison with DDS-cured epoxy resins, DDM-cured FERs exhibited higher contact angle values. The water absorption and contact angle reflect the same patterns, and the DDM-cured FERs exhibited better hydrophobicity. Among them, *d*-FER-DDM showed the highest water contact angle of 108°, implying that the incorporation of trifluoromethyl substitutions makes the materials more hydrophobic and prevents the moisture from being absorbed into epoxy resin [20]. 

### 3.6. Dielectric Properties

**Figure 7:**
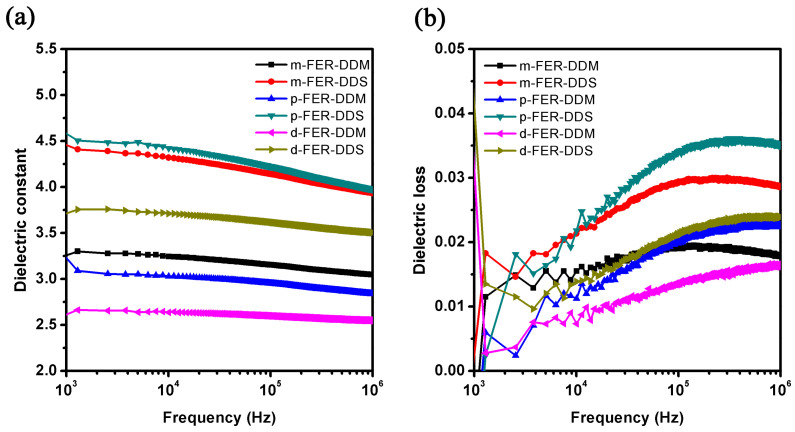
Dielectric properties of fluorinated epoxy resins, (**a**) dielectric constant, (**b**) dielectric loss.

For the microelectronic packaging application, the alternative epoxy resin must possess a low dielectric constant, as well as low dielectric loss, to reduce the delay of signal transmission [12,33]. Figure 7 reveals the relationship of dielectric constant and dielectric loss with the frequency in the range from 1 kHz to 1 MHz at room temperature, and the dielectric constant and dielectric loss of these cured FERs at 1 MHz are displayed in Table 2. As the frequency increased, the dielectric constant of DDS-cured samples decreased slowly, while the dielectric constant of DDM-cured samples remained constant. Meanwhile, the dielectric loss of these cured FERs first increased and then decreased with the frequency increasing. DDM-cured epoxy resins showed a lower dielectric constant than those of DDS-cured samples. This is because the sulfone moiety in DDS results in the higher polarization of cured epoxy resins. The dielectric constant values of these cured FERs are lower than that of commercial DGEBA (3.2~3.6 at 1 MHz) because the −CF_3_ substitutions possess much low polarization and a higher free volume [20]. The cured *d*-FER samples present the lowest dielectric constant due to the incorporation of a higher number of trifluoromethyl substitutions. Among them, the dielectric constant and dielectric loss of *d*-FER-DDM are as low as 2.55 and 0.016 at 1 MHz, respectively. These results indicate that these cured FERs have great potential for applications in electronic packaging and microelectronics.

## 4. Conclusions

A series of epoxy resins with various locations and numbers of trifluoromethyl substitutions were synthesized via a two-step reaction and thermally cured with aromatic amine DDM and DDS. The performance of cured epoxy resins was affected by the molecule structure of epoxy resins and curing agents. The obtained epoxy resins exhibited high glass transition temperatures as well as low water absorption and dielectric constant. The cured FERs also displayed good mechanical properties and high thermal stability. Especially, DDM-cured *d*-FER with two −CF_3_ substitutions on the phenyl side chain exhibited a large water contact angle of 108°, a low water absorption of 0.49%, a low dielectric constant of 2.55, and dielectric loss of 0.016 at 1 MHz. In summary, the incorporation of trifluoromethyl groups into the side chain of epoxy resins can improve the hydrophobic characteristics, moisture resistance, and dielectric properties of cured epoxy resins. Combing their good thermal and mechanical properties, epoxy resins containing pendent trifluoromethyl groups, especially DDM-cured *d*-FER, show great prospects in matrix materials and applications in microelectronics.

## Figures and Tables

**Table 1:** Thermal properties of fluorinated epoxy resins.

Sample	*T*_g_ (°C)	*T*_d5_ (°C)	*T*_d10_ (°C)
*m*-FER-DDM	128	307	332
*m*-FER-DDS	160	316	337
*p*-FER-DDM	147	315	325
*p*-FER-DDS	185	324	342
*d*-FER-DDM	133	326	334
*d*-FER-DDS	178	341	352

**Table 2:** Flexural properties, water absorption, and dielectric properties of fluorinated epoxy resins.

Samples	Flexural Strength (MPa)	Flexural Modulus (GPa)	Water Absorption (%)	Dielectric Constant (1 MHz)	Dielectric Loss (1 MHz)
*m*-FER-DDM	151.36 ± 13.86	2.65 ± 0.23	0.95	3.05	0.018
*m*-FER-DDS	56.46 ± 2.55	1.47 ± 0.31	1.25	3.93	0.029
*p*-FER-DDM	95.55 ± 6.23	1.71 ± 0.34	0.87	2.84	0.023
*p*-FER-DDS	42.30 ± 9.90	1.98 ± 0.06	1.18	3.98	0.035
*d*-FER-DDM	127.00 ± 2.08	2.28 ± 0.37	0.49	2.55	0.016
*d*-FER-DDS	118.85 ± 2.52	1.54 ± 0.27	0.99	3.51	0.024

## Data Availability

All data from the study are included in the article.

## Supplementary Materials

The following supporting information can be downloaded at: https://www.mdpi.com/article/10.3390/polym15132853/s1, Table S1: Curing characteristics of fluorinated epoxy resins; Figure S1: TGA curves of cured FERs.
