# Supplementary material for: Comparison of the Properties of Epoxy Resins Containing Various Trifluoromethyl Groups with Low Dielectric Constant
Source: Polymers (Basel). 2023 Jun 28;15(13):2853. doi: 10.3390/polym15132853 (PMC10346801; doi:10.3390/polym15132853)
Supplement: Supplementary file 1 [file polymers-15-02853-s001.zip › polymers-2465583-supplementary.pdf]

polymers

MDPI

Supplementary Materials

Comparison of the Properties of Epoxy Resins Containing Various Trifluoromethyl Groups with Low Dielectric Constant

Yurong Zhang [1], Haidan Lin [2], Kai Dong [1], Shasha Tang [1] and Chengji Zhao [1,*]

[1] Key Laboratory of High Performance Plastics, Ministry of Education, College of Chemistry, Jilin University, Changchun 130012, China
[2] Electric Power Research Institute, State Grid Jilin Electric Power Company, Changchun 130012, China
* Correspondence: zhaochengji@jlu.edu.cn; Tel.: +86-431-85168870

Table S1. Curing characteristics of fluorinated epoxy resins.

Heating rate (°C/min)	T_i (°C)	T_p (°C)	T_e (°C)	Heating rate (°C/min)	T_i (°C)	T_p (°C)	T_e (°C)
m-FER-DDM				*m*-FER-DDS			
5.0	66.5	137.4	179.8	5.0	142.7	207.6	256.3
7.5	100.3	144.9	192.3	7.5	148.30	219.89	263.56
10.0	109.7	157.6	195.8	10.0	156.27	227.61	271.05
12.5	109.9	162.3	206.3	12.5	163.85	235.91	277.12
15.0	110.9	171.0	209.5	15.0	170.53	239.63	284.04
p-FER-DDM				*p*-FER-DDS			
5.0	102.3	144.4	172.0	5.0	150.7	209.1	252.4
7.5	105.4	155.4	187.1	7.5	153.0	219.1	261.0
10.0	106.6	162.0	194.9	10.0	158.0	224.9	268.8
12.5	108.2	167.2	202.0	12.5	161.9	232.8	273.8
15.0	110.1	172.7	209.4	15.0	164.6	239.5	286.2
d-FER-DDM				*d*-FER-DDS			
5.0	99.3	140.4	205.3	5.0	170.8	215.7	267.2
7.5	102.5	152.5	218.4	7.5	174.0	228.0	277.5
10.0	106.2	161.8	225.1	10.0	176.1	236.8	284.9
12.5	108.2	167.6	235.5	12.5	178.5	244.2	288.2
15.0	109.2	173.2	241.9	15.0	181.6	250.6	294.8

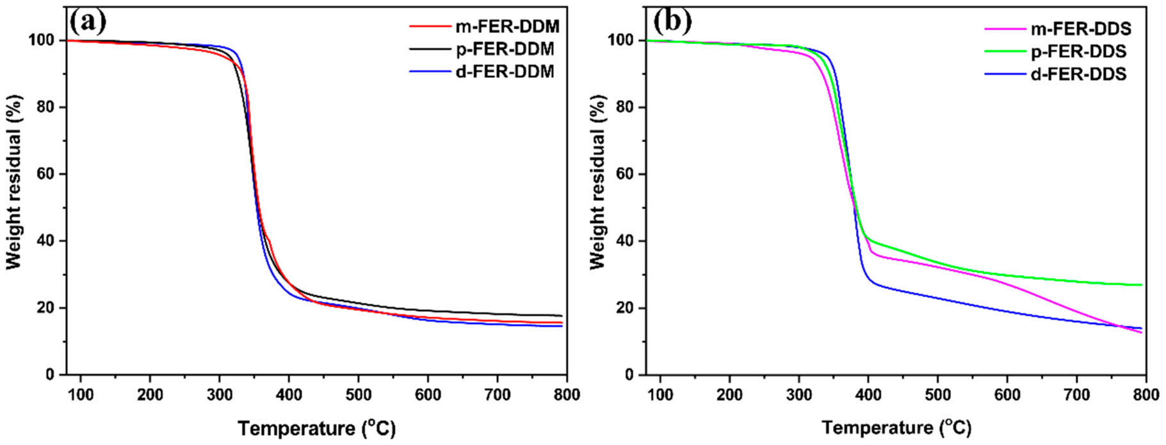

Figure S1. TGA curves of cured FERs.
